# Multidimensional Predictors of Tirzepatide Efficacy: Clinical, Genetic, and Molecular Biomarkers for Glycemic, Weight, and Organ Protection

**DOI:** 10.3390/ph19050791

**Published:** 2026-05-19

**Authors:** Min Hyeok Shin, Jin Woo Jeong, Se Eun Ha, Rajan Singh, Moon Young Lee, Seungil Ro, Tae Yang Yu

**Affiliations:** 1 Division of Endocrinology and Metabolism, Department of Medicine, Wonkwang University Hospital, Wonkwang University School of Medicine, Iksan 54538, Republic of Korea; smh1220@wku.ac.kr (M.H.S.); jinu84@wku.ac.kr (J.W.J.); 2 Department of Physiology and Cell Biology, University of Nevada School of Medicine, Reno, NV 89557, USA; seeunh@med.unr.edu (S.E.H.); rajans@med.unr.edu (R.S.); 3 Department of Physiology, Wonkwang Digestive Disease Research Institute & Institute of Wonkwang Medical Science, Wonkwang University School of Medicine, Iksan 54538, Republic of Korea; lmy6774@hanmail.net

**Keywords:** tirzepatide, treatment outcome, precision medicine, biomarkers, pharmacogenetics, metabolomics, metabolic diseases

## Abstract

Tirzepatide, a dual glucagon-like peptide-1 (GLP-1) and glucose-dependent insulinotropic polypeptide (GIP) receptor agonist, demonstrates robust efficacy in glycemic control and weight reduction. However, substantial interindividual variability in treatment response is observed in clinical practice. In this narrative review, we summarize current evidence on clinical, genetic, and molecular predictors of tirzepatide response and discuss their implications for a precision medicine framework. Data from pivotal clinical trials, post hoc analyses, and relevant preclinical and clinical studies were evaluated to identify determinants of glycemic and weight loss responses, as well as hepatic and renal protective effects. Key clinical predictors include tirzepatide dose, duration of diabetes, β-cell function, baseline glycated hemoglobin, sex, age, race, concomitant therapies, and early treatment response. Genetic factors implicated in treatment variability include variants in GLP-1 receptor, GIP receptor, β-arrestin 1, transcription factor 7-like 2, fat mass and obesity-associated protein, and melanocortin 4 receptor, although tirzepatide-specific validation remains limited. Molecular biomarkers such as branched-chain amino acids, insulin-like growth factor–binding protein-1 and -2, the adiponectin-to-leptin ratio, high-sensitivity C-reactive protein, and interleukin-6 show potential as pharmacodynamic indicators of metabolic response. For organ-specific outcomes, procollagen type III N-terminal peptide and magnetic resonance imaging–proton density fat fraction are supported for assessing hepatoprotective effects, while cystatin C–based estimated glomerular filtration rate and urine albumin-to-creatinine ratio are validated markers of renoprotection. Additional candidates—including tumor necrosis factor receptor 1/2, kidney injury molecule-1, and neutrophil gelatinase-associated lipocalin—are promising but require prospective validation. Overall, predicting response to tirzepatide’s multifaceted therapeutic effects necessitates an integrated, multidimensional approach that incorporates clinical characteristics, genetic variation, and molecular profiling. Ongoing validation and harmonization of these predictors may help establish a precision medicine framework for optimizing tirzepatide therapy.

## 1. Introduction

Type 2 diabetes mellitus (T2DM) is a chronic metabolic disorder characterized by insulin resistance and progressive β-cell dysfunction, with a steadily increasing global prevalence [1]. Conventional glucose-lowering therapies—including metformin, sulfonylureas, and insulin—are effective for glycemic control; however, many older agents were limited not only by adverse effects such as weight gain and hypoglycemia but also by their relatively modest impact on durable improvements in diabetes-related morbidity and mortality [2]. Since the 2000s, the emergence of glucagon-like peptide-1 receptor agonists (GLP-1RAs), which recapitulate the actions of the incretin hormone GLP-1, has introduced a new therapeutic paradigm that enables both glycemic control and weight reduction. In addition, robust evidence has demonstrated cardiovascular benefits, establishing GLP-1RAs as a cornerstone of contemporary clinical practice guidelines [3,4].

Tirzepatide is a dual incretin receptor agonist that activates both glucagon-like peptide-1 (GLP-1) and glucose-dependent insulinotropic polypeptide (GIP) receptors. It is a 39–amino acid synthetic peptide derived from the native GIP sequence, conjugated to a C20 fatty diacid moiety that facilitates albumin binding, thereby extending its half-life to approximately 5 days and enabling once-weekly subcutaneous administration [5].

Pharmacodynamically, tirzepatide exhibits GIP receptor binding affinity comparable to native GIP, while its affinity for the GLP-1 receptor is approximately one-fifth that of native GLP-1. Nevertheless, the combined and complementary activation of both receptors results in synergistic effects, producing greater reductions in glycemia and body weight than those achieved with existing GLP-1RAs [5,6].

Supported by robust clinical trial evidence demonstrating its efficacy, tirzepatide received U.S. Food and Drug Administration approval for the treatment of T2DM in 2022, followed by approval in November 2023 as a therapy for obesity irrespective of diabetes status. Despite its high cost, tirzepatide has been rapidly adopted and is now widely used in clinical practice worldwide.

Nevertheless, despite the consistently robust mean efficacy observed across clinical trials, substantial interindividual variability in response to tirzepatide is evident in both controlled and real-world settings. In some patients (“responders”), treatment leads to pronounced reductions in hemoglobin A1c (HbA1c) and body weight, accompanied by improvements in cardiometabolic risk profiles. In contrast, others (“non-responders” or delayed responders) exhibit attenuated glycemic or weight responses, or require prolonged treatment to achieve clinically meaningful effects.

Post hoc analyses of the SURPASS 1–4 trials suggest that multiple factors may contribute to this variability, including age, sex, duration of diabetes, baseline metabolic status, and early changes in body weight, all of which appear to influence the magnitude of HbA1c reduction and weight loss [7].

Given the substantial cost and expanding cardiometabolic indications of tirzepatide, a clearer understanding of treatment heterogeneity may help clinicians set realistic expectations, interpret early response trajectories, and identify when dose titration, extended observation, or alternative strategies should be considered. Importantly, the factors reviewed here are not intended to support withholding tirzepatide from otherwise appropriate candidates, but rather to facilitate more informed monitoring and individualized follow-up after treatment initiation.

Such a stratified approach may help reduce adverse effects, lower the risk of premature discontinuation, and improve long-term adherence at the patient level. However, it should be regarded as complementary to, rather than a replacement for, pragmatic clinical titration and trial-and-error decision-making. At present, these factors are best applied to contextualize expected variability in response and to guide follow-up intensity, dose titration, and interpretation of early on-treatment changes, rather than to enable definitive pre-treatment patient selection.

**Figure 1:**
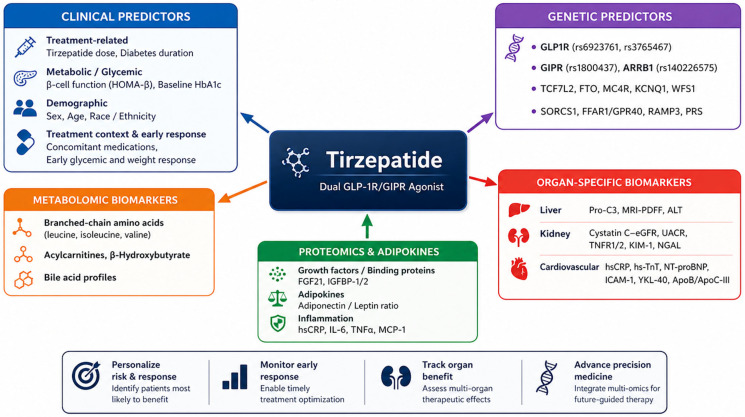
Conceptual Framework of Tirzepatide Response Predictors. A multidimensional model integrates clinical, genetic, metabolic, organ-specific, and proteomic and adipokine predictors. Abbreviations: ALT, alanine aminotransferase; ApoB, apolipoprotein B; ApoC-III, apolipoprotein C-III; eGFR, estimated glomerular filtration rate; FFAR1, free fatty acid receptor 1; FGF21, fibroblast growth factor 21; FTO, fat mass and obesity-associated protein; GIPR, glucose-dependent insulinotropic polypeptide receptor; GLP1R, glucagon-like peptide-1 receptor; HbA1c, hemoglobin A1c; HOMA-β, homeostatic model assessment of β-cell function; hsCRP, high-sensitivity C-reactive protein; hs-TnT, high-sensitivity troponin T; ICAM-1, intercellular adhesion molecule-1; IGFBP, insulin-like growth factor-binding protein; IL-6, interleukin-6; KCNQ1, potassium voltage-gated channel subfamily Q member 1; KIM-1, kidney injury molecule-1; MC4R, melanocortin 4 receptor; MCP-1, monocyte chemoattractant protein-1; MRI-PDFF, magnetic resonance imaging–proton density fat fraction; NGAL, neutrophil gelatinase-associated lipocalin; NT-proBNP, N-terminal pro-B-type natriuretic peptide; PRS, polygenic risk scores; Pro-C3, N-terminal propeptide of type III collagen; RAMP3, receptor activity-modifying protein 3; SORCS1, sortilin-related VPS10 domain containing receptor 1; TCF7L2, transcription factor 7-like 2; TNF-α, tumor necrosis factor-alpha; TNFR1/2, tumor necrosis factor receptor 1/2; UACR, urine albumin-to-creatinine ratio; WFS1, wolframin ER transmembrane glycoprotein; YKL-40, chitinase-3-like protein-1.

Accordingly, this narrative review summarizes and critically appraises the current evidence on clinical, genetic, and molecular predictors of tirzepatide response, with explicit attention to the strength and directness of the evidence supporting each predictor. Although many relevant observations overlap with the broader incretin class, tirzepatide warrants separate consideration because it combines GLP-1 and GIP receptor agonism, demonstrates distinct efficacy profiles across obesity-, diabetes-, hepatic-, renal-, and cardiovascular-related outcomes, and has generated a growing body of tirzepatide-specific post hoc and biomarker evidence. We further highlight predictors with immediate clinical applicability and identify key gaps requiring future investigation. Through this integrated approach, we seek to establish a foundation for precision medicine that enables individualized and optimized use of tirzepatide. The overall conceptual framework of response predictors discussed in this review is illustrated in Figure 1.

## 2. Methods

This is a narrative review. We searched PubMed, MEDLINE and Google scholar for studies published up to March 2026, using combinations of the following terms: “tirzepatide”, “GLP-1 receptor agonist”, “SURPASS”, “SURMOUNT”, “SUMMIT”, “SYNERGY-NASH”, “TREASURE-CKD”, together with “predictor”, “biomarker”, “genetic”, “metabolomics”, “proteomics”, “adipokine”, “renal outcome”, “hepatic outcome”, and “cardiovascular outcome”. Reference lists of included articles and recent review papers were also hand-searched. Preclinical rodent studies are cited throughout this review to provide mechanistic context; however, species differences in incretin biology, adipose tissue distribution, bile acid metabolism, and inflammatory responses limit direct translation to humans. Accordingly, rodent findings are presented as supportive mechanistic evidence rather than as evidence of clinical predictive performance in patients. We prioritized (i) pivotal phase 2/3 trials of tirzepatide and their post hoc analyses, (ii) clinical data on GLP-1 receptor agonists when tirzepatide-specific evidence was unavailable, and (iii) mechanistic and preclinical studies for biomarkers lacking clinical-response data. Articles not published in English were excluded. As this is a narrative review, a formal PRISMA flow and quantitative risk-of-bias assessment were not performed. Instead, the level of evidence for each predictor is explicitly categorized using a pre-specified three-tier framework:

- Tier 1: Direct tirzepatide clinical evidence.

Defined by ≥1 peer-reviewed phase 2 or 3 tirzepatide trial (including pre-specified or post hoc analyses) demonstrating that the biomarker or clinical factor is an independent predictor of response, or exhibits a statistically significant change concordant with the outcome of interest.

- Tier 2: Strong disease-domain evidence requiring tirzepatide-specific validation.

Supported by consistent findings from randomized trials, meta-analyses, or pharmacogenomic studies involving other GLP-1 receptor agonists or within the target disease domains (T2DM, obesity, DKD, MASH, HFpEF), but lacking direct tirzepatide-specific clinical data.

- Tier 3: Mechanistic or preclinical rationale with limited clinical support.

Based primarily on preclinical studies, physiological plausibility, or small uncontrolled clinical reports, with no available human response data for tirzepatide.

## 3. Interindividual Variability in Tirzepatide Response: Responders and Non-Responders

Tirzepatide has demonstrated robust HbA1c-lowering efficacy in patients with T2DM through the SURPASS trials and its weight-reduction efficacy and safety in adults with or without diabetes in the SURMOUNT trials [8,9]. However, not all patients achieve recommended therapeutic targets. Across the SURPASS 1–5 studies, the proportion of participants who did not reach an HbA1c < 6.5% at weeks 40–52 ranged from approximately 14% to 34%, depending on dose and study population. Similarly, in the SURMOUNT 1–3 trials, 9–21% of participants failed to achieve ≥5% weight loss [8,9,10]. These findings underscore clinically meaningful heterogeneity in treatment response. Patients who achieve substantial reductions in HbA1c and body weight may be classified as responders, whereas those with attenuated or delayed responses can be considered non-responders or slow responders. Post hoc analyses of the SURPASS program further suggest that clinical factors can help predict the likelihood of achieving and sustaining glycemic and weight-loss benefits [7].

Beyond glycemic and weight outcomes, tirzepatide exerts pleiotropic effects across multiple organ systems. Cardiovascular benefits have been demonstrated in dedicated trials, including the SUMMIT study, in which tirzepatide significantly reduced the composite endpoint of cardiovascular death or worsening heart failure compared with placebo. Improvements were also observed in patient-reported outcomes, such as the Kansas City Cardiomyopathy Questionnaire Clinical Summary Score, and in biomarkers of cardiovascular stress and inflammation, including N-terminal pro–B-type natriuretic peptide and high-sensitivity C-reactive protein (hsCRP) [11,12].

Renal protective effects have been supported by post hoc analyses of the SURPASS trials, which showed attenuation of the annual decline in estimated glomerular filtration rate (eGFR) and dose-dependent reductions in urine albumin-to-creatinine ratio (UACR) of approximately 19–26% relative to comparators [13,14]. Consistent findings have also been reported in SURMOUNT populations without diabetes.

Hepatic benefits have been highlighted in the SYNERGY-NASH phase 2 trial, where tirzepatide treatment in patients with non-cirrhotic metabolic dysfunction–associated steatohepatitis (MASH) and moderate-to-severe fibrosis resulted in MASH resolution without fibrosis worsening in 44–62% of participants, significantly exceeding placebo [15]. Improvements were also observed in fibrosis-related endpoints and liver fat content assessed by magnetic resonance imaging–proton density fat fraction (MRI-PDFF). Subgroup analyses further demonstrated favorable changes in noninvasive fibrosis markers (e.g., Pro-C3, ELF score, NIS-4) and inflammatory biomarkers.

Collectively, these findings indicate that tirzepatide induces consistent changes in organ-specific biomarkers, suggesting that its cardiometabolic, renal, and hepatic benefits may be predictable through biomarker-based stratification. Accordingly, this review examines not only predictors of glycemic and weight responses but also emerging organ-specific biomarkers relevant to precision medicine approaches.

## 4. Clinical Predictors of Tirzepatide Response

A post hoc analysis of SURPASS-4 showed that 75–84% of tirzepatide-treated participants achieved HbA1c ≤ 6.5% at week 52. Predictors of achieving this target included higher dose, shorter diabetes duration, lower baseline HbA1c, higher HOMA-β, metformin monotherapy, and absence of albuminuria. Maintenance at year 2 was associated with non-use of sulfonylureas, greater triglyceride reduction, smaller high-density lipoprotein (HDL) increase, and higher low-density lipoprotein (LDL) at week 52 [16]. In pooled SURPASS 1–4 analyses, predictors of ≥15% weight loss included higher dose, female sex, White or Asian race, younger age, lower baseline HbA1c and fasting glucose, lower non-HDL cholesterol, and concomitant metformin use [7].

Except for some outcomes such as HbA1c reduction in SURPASS-1, most studies show that higher and sustained tirzepatide doses produce greater glycemic and weight-lowering effects [8,9,10]. This mirrors findings with earlier GLP-1RAs. Adverse events, particularly gastrointestinal (GI), increase with dose: 39% at 5 mg, 46% at 10 mg, and 49% at 15 mg [17]. These events may limit dose attainment, potentially underestimating efficacy. Mediation analysis indicates that GI adverse events account for <6% of tirzepatide-induced weight loss [18].

Diabetes duration is a strong predictor of glycemic response to tirzepatide. In the SURPASS-4 post hoc analysis, shorter duration independently predicted achieving HbA1c ≤ 6.5% at week 52 [16]. This reflects the natural progression of T2DM, where longer disease duration leads to declining β-cell function and reduced responsiveness to incretin therapies [19]. Tirzepatide enhances β-cell function via dual GIP and GLP-1 receptor stimulation—promoting glucose-dependent insulin secretion, lowering the proinsulin/C-peptide ratio, and increasing homeostatic model assessment 2 of β-cell function (HOMA2-β) [6,20]. These effects are meaningful only when sufficient β-cell function remains, so shorter diabetes duration allows greater drug responsiveness [19,21]. This aligns with lifestyle intervention studies, such as DiRECT, where diabetes remission was higher in patients with disease duration ≤ 6 years [22].

β-cell function, while related to diabetes duration, independently predicts response. In SURPASS-4, higher baseline HOMA-β increased the likelihood of achieving HbA1c ≤ 6.5% at week 52, and higher HOMA-β at week 52 predicted glycemic maintenance through year 2 [16]. GLP-1RA studies similarly show that higher fasting C-peptide levels and postprandial urinary C-peptide/creatinine ratios predict HbA1c response [23], highlighting β-cell reserve as a key determinant. SURMOUNT-2 data suggest a bidirectional relationship: patients with higher β-cell function achieve greater weight loss, whereas those with lower function have larger absolute HbA1c reductions [24], reflecting differing primary pathways—weight loss/insulin resistance improvement versus glycemic reduction.

Baseline HbA1c predicts both glycemic and weight responses. In SURPASS-4, lower baseline HbA1c independently predicted achieving HbA1c ≤ 6.5% at week 52 [16], and pooled SURPASS 1–4 analyses confirmed it as a predictor of ≥15% weight loss [7,25]. While lower baseline HbA1c favors target attainment, higher baseline values yield greater absolute HbA1c reduction, a pattern seen with GLP-1RAs [26,27]. Lower baseline HbA1c may also indicate better metabolic health, lower insulin resistance, and less use of weight-promoting drugs (e.g., sulfonylureas and insulin) [7,16], consistent with greater weight loss observed in obese patients without diabetes (SURMOUNT-1: ~22.5% at 15 mg vs. 9–14% in SURPASS 1–5) [8,9].

Sex is a strong predictor of weight loss with tirzepatide. In pooled SURPASS 1–4 analyses, women were ~2.6 times more likely than men to achieve ≥15% weight loss [7], and in SURPASS-4, female sex was the strongest predictor of ≥10% weight loss [16]. This pattern is consistent across other GLP-1RAs, with a meta-analysis showing women lost on average 0.88 kg more than men, with the difference increasing at higher weight loss levels [28].

Several mechanisms may explain this sex difference. First, women typically have lower body weight and drug clearance, leading to higher systemic exposure to GLP-1RAs [29,30]. Second, estrogen enhances GLP-1 signaling, increasing suppression of food reward; blocking estrogen receptors reduces this effect, and responses are greater in female vs. male rodents [31]. Third, more frequent GI adverse events in women may reduce intake [29] but their mediation of weight loss is <6%, insufficient to fully explain the difference [18].

Age modestly affects weight loss with tirzepatide. In pooled SURPASS 1–4 analyses, each 5-year increase reduced the likelihood of ≥15% weight loss [7], and early fasting glucose responders were generally younger [32]. A blunted response in older patients may reflect lower metabolic rate, reduced muscle mass and physical activity, and age-related declines in incretin and β-cell function. However, the effect is modest, and age alone should not be used to restrict tirzepatide use.

Race and ethnicity may influence weight loss response. In pooled SURPASS 1–4 analyses, White and Asian individuals were more likely to achieve ≥15% weight loss [7]. The mechanisms are unclear but may involve differences in body composition, genetic variants affecting drug metabolism, and lifestyle factors.

Concomitant therapy affects tirzepatide response. Patients on metformin monotherapy achieved higher rates of glycemic targets [16] and concomitant metformin use independently predicted ≥15% weight loss [7]. This likely reflects earlier disease stage, preserved β-cell function, and absence of weight-promoting drugs (e.g., sulfonylureas and insulin). In contrast, concurrent sulfonylurea use at week 52 predicted failure to maintain glycemic control at year 2 [16], likely due to glucose-independent insulin secretion accelerating β-cell exhaustion and reducing long-term incretin efficacy [33,34].

Early response may predict long-term efficacy. In post hoc analyses from the SURPASS trials, patients achieving ≥20% reductions in fasting glucose at week 4 or ≥5% weight loss at week 8 experienced greater HbA1c lowering, weight reduction, and cardiometabolic improvements by weeks 40–42 [32]. These early indicators can inform individualized management. In early responders, the current dose can be maintained with routine titration toward the target, avoiding premature escalation that may increase gastrointestinal adverse effects. In early non-responders, options include extending the titration interval, escalating to the next dose level as tolerated, reinforcing lifestyle interventions, or—if HbA1c remains suboptimal despite full-dose therapy—considering addition or transition to alternative agents. Notably, a blunted early response alone should not prompt discontinuation, as many slower responders still achieve meaningful benefit with continued treatment.

In summary, tirzepatide’s glycemic and weight responses are influenced by dose, diabetes duration, β-cell function, baseline HbA1c, sex, age, race, and concomitant therapy. These predictors help estimate individual responses and guide treatment goals, with early responses at weeks 4–8 providing practical indicators for therapy adjustment. While these clinical factors are readily assessed, emerging genetic and molecular markers may further refine prediction. Clinical predictors of tirzepatide response are summarized in Table 1.

## 5. Genetic Predictors

The pharmacological response to tirzepatide may be modulated by genetic variation at multiple levels, including receptor binding, intracellular signal transduction, and downstream metabolic effector pathways. Emerging evidence has identified specific loci and polygenic profiles that may help predict interindividual variability in treatment response [35]. Table 2 summarizes the key genetic predictors of tirzepatide response. A limitation is that most currently available genetic evidence is derived from GLP-1RA pharmacogenetic or mechanistic studies rather than tirzepatide-specific human cohorts.

Variants in the *GLP1R* gene are biologically plausible determinants of tirzepatide response, as their effects on insulin secretion, glucagon suppression, and appetite are mediated through the GLP-1 receptor. The missense variant rs6923761 (G168S) has been linked to greater HbA1c reduction and weight loss [36], with the Ser168 allele enhancing insulin secretory responses to GLP-1 [37]. Another variant, rs3765467, may alter ligand binding and has been associated with early-onset T2DM [38] and greater HbA1c reduction with GLP-1RAs [39]. These findings suggest that *GLP1R* variants may modulate the magnitude of therapeutic response.

Variants in the *GIPR* gene may influence tirzepatide response by modulating GIPR–mediated signaling. These variants are associated with reduced GIP-stimulated insulin secretion and diminished incretin activity [40]. The rs1800437 (E354Q) variant impairs receptor signaling and lowers C-peptide levels [41], while loss-of-function variants such as rs139215588 (R190Q) and rs143430880 (E288G) are linked to lower BMI [42]. Although these changes could attenuate tirzepatide’s weight-loss effects, overall signaling remains largely preserved, with only modest reductions in downstream pathways such as inositol monophosphate accumulation and AKT phosphorylation [43].

Beta-arrestin 1 (ARRB1) regulates GLP-1R desensitization while mediating signaling pathways that promote cAMP production, ERK/CREB activation, and insulin secretion in β-cells [44]. A rare variant, rs140226575, has been associated with enhanced HbA1c reduction (~0.6–0.8%) in response to GLP-1RAs [45].

Transcription factor 7-like 2 (TCF7L2) regulates β-cell function and incretin response via Wnt signaling, and its variants are linked to T2DM risk [46]. The rs7903146 T allele is associated with impaired incretin-stimulated insulin secretion, with TT carriers showing reduced GLP-1–mediated effects [47,48]. However, recent data have not confirmed a clear association with GLP-1RA response, and its role in tirzepatide efficacy remains uncertain [49].

Variants in the fat mass and obesity-associated (*FTO*) gene are associated with obesity-related traits, including appetite regulation, energy balance, and adiposity. The rs9939609 A allele is linked to higher BMI and increased appetite [50]. Experimental data suggest exenatide can suppress high-fat diet–induced *FTO* expression [51], while *FTO* risk variants may impair central satiety signaling and increase food intake, potentially attenuating the weight-loss response to tirzepatide [52].

Melanocortin 4 receptor (MC4R) is a key regulator of appetite and energy balance, and loss-of-function variants are a common cause of monogenic obesity, characterized by hyperphagia and early-onset weight gain [53,54]. Despite this, studies in *MC4R* mutation carriers show that GLP-1RAs and tirzepatide achieve weight loss comparable to controls [55,56]. Current evidence suggests minimal impact on tirzepatide efficacy, though larger studies are needed.

Potassium voltage-gated channel subfamily Q member 1 (KCNQ1) is involved in pancreatic β-cell insulin secretion. Its common variants, rs2237892 and rs2237895, are strongly associated with T2DM in East Asian populations and have been linked to impaired insulin secretion [57]. These findings suggest that tirzepatide’s GLP-1– and GIP–mediated effects may vary by genotype; however, evidence remains inconsistent, particularly regarding associations with GLP-1 levels [58,59].

Wolframin ER transmembrane glycoprotein (*WFS1*) encodes an endoplasmic reticulum protein involved in stress response and calcium homeostasis and is the causative gene for Wolfram syndrome, characterized by diabetes insipidus, diabetes mellitus, optic atrophy, and deafness [60]. Common variants such as rs10010131 and rs734312 are associated with T2DM risk and impaired β-cell function [61,62]. Given its role in insulin processing and secretion, *WFS1* variants may influence incretin-stimulated insulin responses. Notably, case reports suggest that GLP-1RA therapy can markedly improve glycemic control in Wolfram syndrome, in some cases allowing insulin discontinuation [63,64].

Sortilin-related VPS10 domain-containing receptor 1 (SORCS1) regulates insulin granule trafficking and processing. Its variants are associated with T2DM risk and impaired insulin secretion [65]. Experimental models show disrupted islet morphology and reduced insulin content, possibly related to effects on islet microvasculature via platelet-derived growth factor signaling [66]. These findings suggest that incretin-stimulated insulin secretion may depend on SORCS1 function. Consistently, stratified analyses by rs1416406 genotype in exenatide-treated patients demonstrate differences in HbA1c, glycemia, and β-cell function [67,68].

Free fatty acid receptor 1 (*FFAR1*) encodes G protein–coupled receptor 40 (GPR40), which enhances fatty acid–stimulated insulin secretion in pancreatic β-cells and mediates incretin release. GPR40-deficient mice show reduced GLP-1 and GIP secretion, indicating that FFAR1 variants may influence endogenous incretin levels [69]. GPR40 also modulates food intake and body weight via GLP-1, and although drug development targeting GPR40 was halted due to hepatotoxicity [70], *FFAR1* variants remain relevant for understanding individual sensitivity to the incretin system.

Receptor activity–modifying proteins (RAMPs) regulate agonist binding, signal transduction, cell surface expression, and signaling bias of drug targets. Among them, RAMP3 interacts with the GLP-1 receptor. In animal studies, *RAMP3* overexpression increased GLP-1 sensitivity and glucose-stimulated insulin secretion, while reduced expression decreased responsiveness [71]. Although the effects of *RAMP* variants on GLP-1RA efficacy remain unclear, they may modulate tirzepatide’s dual-receptor activity and warrant investigation in future pharmacogenomic studies.

To address the limited impact of individual genetic variants, polygenic risk scores (PRS)—which integrate multiple variants—have emerged as a promising tool for predicting tirzepatide response. Meta-analyses of genome-wide association studies (GWAS) have linked variants in several of the genes discussed above to glycemic responses to GLP-1RAs [35]. Additionally, GLP-1RA therapy has been associated with greater weight loss in individuals with high obesity PRS [72], whereas adults with T2DM and high BMI PRS showed slightly reduced weight loss over 52 weeks [73].

These considerations should, however, be tempered by recent large-scale genetic evidence. Population-level analyses—including recent genome-wide and whole-exome studies—indicate that coding variants in *GLP1R* are associated with only modest changes in BMI and glycemia, while loss-of-function variants in *GIPR* are primarily linked to increased gastrointestinal adverse effects rather than reduced incretin efficacy [74]. Thus, although pharmacogenetic variation remains biologically plausible as a modifier of tirzepatide response, effect sizes are generally small, allele frequencies are often low, and their contribution to individual-level prediction—without a validated polygenic score in tirzepatide-treated cohorts—appears limited. Accordingly, current evidence does not support routine clinical use of these variants as predictive biomarkers of tirzepatide response. Further studies are needed to determine whether genetic information can ultimately contribute to clinically meaningful prediction of treatment response.

**Table 2:** Genetic variants associated with response to incretin-based therapies. The Evidence Source column specifies whether each association is derived from tirzepatide clinical data, clinical evidence from GLP-1 receptor agonists requiring tirzepatide-specific validation, or preclinical, physiological, or case-report evidence.

Gene	Variant (RefSNP cluster ID)	Functional Impact	Reported Association	Evidence Source	References
*GLP1R*	rs6923761 (Gly168Ser)	Affects insulin secretory response to GLP-1 during oral glucose challenge	Greater HbA1c and weight reduction with GLP-1RA	GLP-1RA clinical	[36,37]
*GLP1R*	rs3765467	Modifies ligand binding affinity; associated with early-onset T2DM risk	Greater HbA1c reduction with exenatide and liraglutide	GLP-1RA clinical	[38,39]
*GIPR*	rs1800437 (Glu354Gln)	Reduces GIP receptor signaling efficiency	Lower fasting and post-OGTT C-peptide; reduced incretin effect; no significant impact on weight-loss, but increased nausea/vomiting in tirzepatide-treated individuals	Physiological; tirzepatide in vitro; GLP-1RA GWAS	[40,41,42,43,74]
*ARRB1*	rs140226575	Regulates GLP-1R desensitization, internalization, and β-arrestin–dependent signaling	Additional HbA1c reduction (~0.6–0.8%) with GLP-1RA	GLP-1RA GWAS	[44,45]
*TCF7L2*	rs7903146	Impaired incretin-mediated insulin secretion via Wnt signaling	Reduced GLP-1–stimulated insulin secretion (TT genotype)	Physiological; GLP-1RA (inconclusive)	[46,47,48,49]
*FTO*	rs9939609	Alters CNS satiety signaling; associated with increased appetite and BMI	Potentially attenuated weight loss response to tirzepatide	Preclinical/physiological	[50,51,52]
*MC4R*	Loss-of-function variants	Causes hyperphagia and early-onset obesity	Comparable weight reduction with GLP-1RA and tirzepatide versus controls	Tirzepatide clinical	[53,54,55,56]
*KCNQ1*	rs2237892, rs2237895	Impairs insulin secretion via voltage-gated K^+^ channel	Strong association with T2DM (especially in East Asians); inconsistent effects on GLP-1 level	Physiological	[57,58,59]
*WFS1*	rs10010131, rs734312	Impairs β-cell function; insulin processing, and ER stress responses	Associated with T2DM susceptibility; GLP-1RA improved glycemic control in Wolfram syndrome	GLP-1RA case report	[60,61,62,63,64]
*SORCS1*	rs1416406	Disrupts islet architecture and insulin granule trafficking (via PDGF-related pathways)	Genotype-dependent differences in HbA1c, glucose, and β-cell function with exenatide	GLP-1RA clinical	[65,66,67,68]
*FFAR1 (GPR40)*	—	Mediates FFA-stimulated incretin (GLP-1/GIP) secretion	Reduced GLP-1 and GIP secretion in GPR40-deficient mice	Preclinical	[69,70]
*RAMP3*	—	Modulates GLP-1R surface expression and signaling bias	Enhanced GLP-1–mediated insulin secretion with RAMP3 overexpression	Preclinical	[71]

Abbreviations: ARRB1, beta-arrestin 1; BMI, body mass index; CNS, central nervous system; ER, endoplasmic reticulum; FFA, free fatty acid; FFAR1, free fatty acid receptor 1; FTO, fat mass and obesity-associated protein; GIP, glucose-dependent insulinotropic polypeptide; GIPR, GIP receptor; GLP-1, glucagon-like peptide-1; GLP-1RA, GLP-1 receptor agonist; GLP1R, GLP-1 receptor; GPR40, G protein-coupled receptor 40; GWAS, genome-wide association study; HbA1c, hemoglobin A1c; KCNQ1, potassium voltage-gated channel subfamily Q member 1; MC4R, melanocortin 4 receptor; OGTT, oral glucose tolerance test; PDGF, platelet-derived growth factor; RAMP3, receptor activity-modifying protein 3; SORCS1, sortilin-related VPS10 domain containing receptor 1; T2DM, type 2 diabetes mellitus; TCF7L2, transcription factor 7-like 2; WFS1, wolframin ER transmembrane glycoprotein.

## 6. Metabolomic Predictors

Metabolomics captures real-time metabolic states beyond genomics or proteomics by profiling numerous small metabolites in biofluids. Because tirzepatide targets multiple pathways—β-cell insulin secretion, insulin sensitivity, fatty acid oxidation, and appetite—via dual GIP/GLP-1 receptors, baseline metabolite profiles may predict treatment response.

Branched-chain amino acids (BCAAs: leucine, isoleucine, valine) are reproducible biomarkers linked to insulin resistance, T2DM, and obesity. In the Framingham Offspring cohort, elevated BCAAs predicted over fivefold higher diabetes risk, persisting up to 12 years [75]. BCAA levels are also 14–20% higher in obesity, and combined high-fat diet and BCAAs worsen insulin resistance. Mechanistically, BCAAs may activate the mTOR/S6K1 pathway and promote IRS-1 serine phosphorylation [76].

Preclinical studies show tirzepatide lowers circulating BCAAs by enhancing their catabolism, potentially improving insulin sensitivity and cardiovascular outcomes via suppression of BCAA/mTOR signaling [77]. BCAA levels also decline with weight loss during GLP-1RA therapy, alongside improved insulin resistance [78].

Importantly, early changes in BCAA levels may reflect pharmacodynamic engagement and may be associated with subsequent metabolic improvements. In a post hoc metabolomics analysis of a tirzepatide phase 2b trial, BCAAs, glutamate, 3-hydroxyisobutyrate, and branched-chain ketoacids decreased by week 4 and continued through weeks 12 and 26 [79]. Early reductions at weeks 4 and 12 correlated with later improvements in HbA1c, HOMA2-IR, and proinsulin, were tirzepatide dose-dependent, and exceeded those observed with dulaglutide [79].

Acylcarnitines, intermediates of mitochondrial fatty acid β-oxidation, reflect oxidative completeness and mitochondrial function. Elevated medium- and long-chain species (C10–C14, C16–C18) are consistently linked to T2DM and insulin resistance, indicating incomplete long-chain fatty acid β-oxidation and reduced TCA cycle activity [80,81,82]. They may act as biomarkers or contributors to insulin resistance via mechanisms such as NF-κB activation [80]. Notably, tirzepatide did not significantly alter short-chain acylcarnitines (C3, C5), suggesting the need to examine its effects on medium- and long-chain species [79].

Beta-hydroxybutyrate (BHB), the main ketone body produced during glucose depletion, provides an alternative energy source during fasting or caloric deficit and modulates gene expression through histone deacetylase inhibition and GPCR signaling [83]. In insulin resistance and metabolic syndrome—such as T2DM and obesity—hepatic ketogenesis is blunted during fasting, while ketone levels remain elevated in the fed state, leading to dampened ketone kinetics and reduced metabolic flexibility [84].

Mildly elevated ketones have been inversely associated with insulin resistance [85] and maintaining BHB > 0.5 mM correlates with greater weight loss in T2DM [86]. Thus, adaptive BHB increases during tirzepatide-induced energy deficit may indicate efficient fatty acid oxidation and greater weight loss. However, whether baseline or early BHB responses predict tirzepatide efficacy remains unclear and requires prospective study.

Bile acids form a metabolic network in which primary bile acids (e.g., cholic acids, CAs; chenodeoxycholic acids, CDCAs) from the liver are converted by gut microbiota into secondary bile acids (e.g., deoxycholic acids, DCAs; lithocholic acids, LCAs). Secondary bile acids, especially LCA and DCA, activate TGR5 to increase GLP-1 secretion, promoting insulin release from pancreatic β-cells. Circulating bile acid profiles are altered in obesity, T2DM, and NAFLD, often preceding disease onset [87]. Fasting bile acids are higher in obesity and T2DM, but postprandial increases are blunted, and elevated taurine-conjugated bile acids associate with higher glucose, HbA1c, and insulin resistance. Microbiota dysbiosis further disrupts secondary bile acid conversion [87,88]. In diabetic mice, tirzepatide shifted bile acid composition toward a metabolically favorable profile, modulated gut microbiota, and improved hepatic steatosis [89]. Thus, favorable bile acid changes after tirzepatide may reflect treatment response, though this remains untested.

Taken together, the metabolomic signals reviewed here do not yet constitute a coherent or reproducible predictive signature. BCAAs represent the most consistent finding across tirzepatide and GLP-1RA studies. In contrast, acylcarnitines, ketone bodies, and bile acid profiles show directionally plausible but inconsistent associations and have not yet been validated in tirzepatide-treated cohorts. Accordingly, the current metabolomic evidence is best interpreted as hypothesis-generating, with BCAA dynamics emerging as the most promising candidate for future pharmacodynamic investigation.

## 7. Proteomics and Adipokine Predictors

Fibroblast growth factor 21 (FGF21) is a liver-derived hormone that signals through the FGFR1–KLB complex, enhancing insulin sensitivity, increasing energy expenditure and weight loss, lowering triglycerides, and suppressing carbohydrate and sugar intake [90]. Despite these benefits, patients with T2DM paradoxically exhibit elevated FGF21 yet reduced responsiveness due to FGF21 resistance, driven by reduced expression of its receptors, FGFR1c and β-Klotho, in visceral adipose tissue, leading to impaired FGF21 signaling [91]. Elevated baseline FGF21 also predicts future T2DM risk [92]. Preclinical studies show GLP-1RAs improve metabolic markers—including FGF21, adiponectin, triglycerides, and leptin—during pregnancy, and that FGF21 partly mediates GLP-1RA–induced weight loss, as liraglutide effects are blunted in liver-specific Fgf21 knockout mice [93,94]. Thus, FGF21 resistance may attenuate response to tirzepatide, although direct clinical evidence remains limited.

Insulin-like growth factor binding proteins (IGFBPs) regulate IGF activity in development, cell proliferation and differentiation, and metabolic processes. IGFBP-1 and IGFBP-2 are closely linked to metabolic disorders, including obesity, metabolic syndrome, insulin resistance, and T2DM [95]. Low IGFBP-1 predicts insulin resistance and future T2DM risk, while IGFBP-2 inversely correlates with BMI, adiposity, insulin levels, and fatty liver index [6,96].

In animal studies, IGFBP-2 overexpression reduces fat accumulation and insulin resistance, suggesting protective metabolic effects [97,98]. In patients with T2DM, tirzepatide and dulaglutide increase IGFBP-1 and IGFBP-2 levels, with tirzepatide showing stronger insulin-sensitizing effects [6]. Thus, IGFBPs may serve as pharmacodynamic biomarkers of improved insulin sensitivity.

Adipose tissue is an active endocrine organ that secretes bioactive factors regulating metabolism and physiology [99]. Leptin, primarily from adipose tissue, regulates food intake, neuroendocrine function, reproduction, angiogenesis, and blood pressure [100]. Adiponectin is also produced almost exclusively by adipose tissue and exerts cardioprotective, insulin-sensitizing, anti-steatotic, and anti-inflammatory effects [101]. In obesity and T2DM, adiponectin levels decrease while leptin increases [102]. Thus, the adiponectin/leptin ratio serves as an indicator of adipose dysfunction and correlates with inflammation, insulin resistance, oxidative stress, and cardiometabolic risk [102]. Tirzepatide improves this ratio by increasing adiponectin and decreasing leptin in association with weight loss [6,103]. Consistently, GLP-1RAs significantly elevate circulating adiponectin levels [104]. An early increase in adiponectin accompanied by reduced leptin may indicate effective adipose remodeling, including improved adipocyte function and reduced visceral fat, and may be associated with sustained improvements in insulin sensitivity and long-term weight loss.

Obesity is closely linked to chronic low-grade inflammation. Hypertrophied adipocytes secrete monocyte chemoattractant protein-1 (MCP-1, aka CCL2), recruiting monocytes that differentiate into pro-inflammatory M1 macrophages producing TNF-α and IL-6, creating a vicious cycle [105,106]. The key regulator is NF-κB, which is activated by TNF-α, free fatty acids, reactive oxygen species, and hypoxia, amplifying MCP-1, TNF-α, and IL-6 expression via a positive feedback loop [107]. This inflammation drives insulin resistance, T2DM, and cardiovascular disease through impaired insulin signaling, hsCRP, and vascular endothelial dysfunction [107].

Given this role, anti-inflammatory effects may reflect tirzepatide efficacy and may serve as pharmacodynamic markers of on-treatment response. A meta-analysis of SURPASS and SURMOUNT trials showed reduced hsCRP and a −17.8% decrease in IL-6 versus placebo [108]. Preclinical studies further show tirzepatide inhibits NF-κB in adipose tissue macrophages, lowering TNF-α and MCP-1 and promoting M1-to-M2 polarization [109,110]. Similarly, GLP-1RAs reduce TNF-α and MCP-1 [109,111], supporting inflammatory cytokines as potential on-treatment biomarkers of tirzepatide response.

## 8. Organ-Specific Markers

The clinical benefits of tirzepatide extend beyond glycemic control and weight loss to the protection of key organs, including the liver, kidneys, and cardiovascular system. Accordingly, the characterization and monitoring of treatment responsiveness should encompass not only HbA1c and weight reduction but also organ-protective effects. This chapter reviews biomarkers that may predict or monitor protection across these organ systems. The biomarkers discussed below are organized according to the strength of available tirzepatide-specific clinical evidence. For several markers, particularly those related to renal tubular injury, direct clinical data in tirzepatide-treated patients are not yet available, and their inclusion reflects pathophysiological rationale and extrapolation from disease-domain evidence rather than validated predictive performance with tirzepatide.

Clinical studies show tirzepatide improves MASH, reducing hepatic steatosis, visceral and saturated fat, and resolving MASH without worsening fibrosis [15,112]. Switching from other GLP-1RAs to tirzepatide also promotes weight loss, improves liver enzymes, and reduces liver fat with histological improvement without fibrosis progression [113]. These findings support robust hepatoprotective effects, with relevant biomarkers for monitoring treatment response outlined below.

Pro-C3, the N-terminal propeptide of type III collagen, reflects extracellular matrix (ECM) formation during hepatic fibrogenesis. Increased collagen (types I, III, IV) deposition releases circulating neo-epitope fragments that indicate ECM remodeling and liver disease activity. Unlike older markers, Pro-C3 reflects collagen formation specifically [114,115]. It independently predicts advanced fibrosis (≥F3) and, within the ADAPT algorithm, shows superior accuracy (AUROC 0.86–0.87) over the Fibrosis-4 Index (FIB-4), APRI, and NAFLD fibrosis score [116]. Meta-analysis confirms good diagnostic performance (AUROC 0.73–0.84 for ≥F2; 0.73–0.89 for ≥F3) [117]. Tirzepatide studies, including SYNERGY-NASH, consistently show reduced serum Pro-C3 in patients with T2DM [15,118].

MRI-PDFF is a quantitative imaging biomarker of liver fat that strongly correlates with histologic steatosis and shows excellent reproducibility, making it a widely used noninvasive endpoint in NAFLD/MASH trials. A multicenter secondary analysis of the FLINT trial identified a ≥30% relative reduction as the optimal cutoff for predicting histologic response [119], and meta-analysis confirmed this threshold is associated with higher odds of histologic improvement and NASH resolution [120]. In the SYNERGY-NASH study, MRI-PDFF reductions at week 52 were −45.7%, −41.3%, and −57.0% with tirzepatide 5/10/15 mg, respectively, exceeding placebo (−9.8%). Additionally, other liver markers—such as circulating miR-122, FIB-4, ELF score, cytokeratin-18, and ALT—exist; however, aside from the ALT reductions observed in the SURPASS studies, direct evidence linking these markers to tirzepatide is lacking [112,121,122].

Tirzepatide’s renal protective effects have been confirmed in multiple large studies. In SURPASS-4, it slowed eGFR decline, reduced UACR, and improved composite renal outcomes in T2DM [14], with similar findings in a pooled SURPASS 1–5 analysis [13]. SURMOUNT post hoc analysis showed greater UACR reduction versus placebo in overweight/obese patients, regardless of diabetes [123]. A meta-analysis of 14,471 participants confirmed UACR benefits without adverse effects on eGFR [124]. The ongoing TREASURE-CKD study is evaluating direct renal effects in overweight/obese patients with chronic kidney disease (CKD) using multiparametric MRI to measure renal oxygenation, sinus fat, blood flow, glomerular filtration rate (GFR), and albuminuria [125]. Candidate biomarkers for patient stratification, early response detection, and monitoring of these renal effects are outlined below.

Cystatin C, a 13 kDa protein produced by all nucleated cells, is freely filtered by the glomerulus and reabsorbed and degraded in the proximal tubule. Unlike creatinine, it is independent of muscle mass, sex, or race, making it a reliable marker of GFR [126,127]. Because tirzepatide can reduce body weight by up to 13.5 kg, associated loss of skeletal muscle may lower serum creatinine, potentially causing creatinine-based eGFR to overestimate actual renal function. In SURPASS-4, cystatin C–based eGFR declined less with tirzepatide (−3.5 mL/min/1.73 m^2^) versus insulin glargine (−5.3 mL/min/1.73 m^2^), consistent with creatinine-based measures, and changes in eGFR were not correlated with weight loss, supporting true renal protection [128].

SURMOUNT-1 showed a 3.2 mL/min/1.73 m^2^ higher cystatin C–eGFR with tirzepatide versus placebo at week 72 in non-diabetic overweight/obese patients [123]. In SUMMIT, tirzepatide increased both cystatin C– and creatinine-based eGFR at week 52, but early discordance suggested cystatin C better reflects renal changes during weight loss [129]. Thus, cystatin C–based eGFR is a valuable complementary biomarker for assessing tirzepatide’s renal protective effects.

UACR is the primary biomarker for diagnosing, staging, and predicting diabetic kidney disease (DKD). A ≥30% reduction in UACR is linked to slower long-term eGFR decline and lower end-stage kidney disease (ESKD) risk [130,131]. It is the most consistently reported renal outcome for tirzepatide. In SURPASS-4, tirzepatide reduced UACR dose-dependently versus insulin glargine, and pooled SURPASS 1–5 analysis showed reductions of −19.3%, −22.0%, and −26.3% with 5/10/15 mg at weeks 40/42, greatest in patients with baseline UACR ≥ 30 mg/g [13]. SURMOUNT analyses reported −8.4% (non-diabetic, SURMOUNT-1) and −31.1% (diabetic, SURMOUNT-2) reductions versus placebo, with −42.3% and −55.2% in baseline UACR ≥ 30 mg/g subgroups [128]. Mediation analysis suggests about half of the UACR reduction is weight loss–dependent, indicating both weight loss–dependent and –independent effects of tirzepatide. Overall, UACR has the strongest evidence for monitoring renal protection, and baseline UACR may help predict treatment response.

Soluble TNF receptors 1 and 2 (TNFR1/2, 55 and 75 kDa) are cleaved from cell membranes and regulate apoptosis, inflammation, and immune responses [132]. Joslin Diabetes Center studies first showed that elevated TNFR1/2 strongly predict DKD progression and ESKD in both type 1 diabetes mellitus (T1DM) and T2DM, independent of proteinuria [132,133]. Meta-analysis reported relative risks of 2.51 for TNFR1 and 3.23 for TNFR2 in DKD progression [134]. In diabetic CKD, TNFR1/2 and soluble urokinase plasminogen activator receptor (suPAR) independently predict all-cause mortality beyond eGFR and UACR [135]. Histologically, TNFR1/2 correlate with glomerular basement membrane thickening, mesangial expansion, podocyte hypertrophy, and glomerulosclerosis, and inversely with endothelial fenestration and filtration surface area, suggesting a direct role in diabetic glomerular structural damage [136]. Preclinical studies suggest GLP-1 receptor activation may suppress renal TNFα pathways through anti-inflammatory effects [137], but no clinical data exist on tirzepatide’s effect on circulating TNFR1/2. Given their predictive value for DKD progression, assessing TNFRs in future trials could clarify tirzepatide’s renal protective mechanisms and inform their potential role as response-monitoring biomarkers. To date, no prospective clinical studies have evaluated changes in circulating TNFR1/2 in tirzepatide-treated patients. Accordingly, TNFR1/2 should be regarded as a candidate biomarker requiring dedicated validation, rather than an established predictor of tirzepatide response.

Kidney Injury Molecule-1 (KIM-1) is a type I transmembrane glycoprotein minimally expressed in normal kidneys but markedly upregulated in proximal tubular epithelial cells after ischemic or toxic injury, and released into blood and urine [138]. It serves as an early marker of acute kidney injury (AKI) and chronic tubulointerstitial damage [139]. In T1DM, higher baseline serum KIM-1 correlated with faster eGFR decline, and 63% of patients above the median (97 pg/mL) reached ESKD versus 20% below [140]. However, its predictive value may be limited when adjusted for albumin excretion [141]. In DKD, KIM-1 is particularly useful for detecting early tubular injury before albuminuria onset [142,143]. Preclinical studies suggest tirzepatide lowers KIM-1 [144], but clinical evidence is limited. Future studies assessing KIM-1 could clarify tirzepatide’s tubular protective effects.

Neutrophil gelatinase-associated lipocalin (NGAL) is a 25 kDa lipocalin protein mainly secreted by neutrophils, macrophages, and dendritic cells in response to inflammation. Kidney injury markedly increases its expression in the distal tubule, releasing it into blood and urine, making it an early AKI biomarker [145,146]. A meta-analysis in DKD reported serum NGAL with 0.79 sensitivity and 0.87 specificity, and urinary NGAL with 0.85 sensitivity and 0.74 specificity [147]. Notably, in normoalbuminuric diabetic patients, serum NGAL showed 0.90 sensitivity, 0.97 specificity, and AUC 0.973, detecting tubular injury before albuminuria [147]. In CKD, serum and urinary NGAL correlate with disease severity and predict renal function decline [146]. While no clinical data link tirzepatide directly to NGAL, GLP-1RAs reduce tubular injury via anti-oxidative and anti-inflammatory effects in preclinical models [137]. Given species differences in tubular physiology between rodents and humans, these preclinical findings cannot be directly extrapolated to patients. Nevertheless, they support NGAL as a potential biomarker for evaluating tirzepatide-associated tubular protective effects in future clinical studies.

Imaging biomarkers can noninvasively assess structural and functional organ changes. In the kidney, multiparametric MRI can simultaneously measure oxygenation (R2*/BOLD), fibrosis (T1 mapping), perfusion (arterial spin labeling), and microstructure (diffusion-weighted imaging), capturing the multidimensional pathophysiology of kidney disease [148]. The TREASURE-CKD study (NCT05536804) currently evaluates tirzepatide’s renal effects in overweight/obese patients with CKD using MRI parameters such as renal oxygenation, sinus fat, blood flow, GFR, and albuminuria [125]. This approach directly visualizes changes beyond conventional biomarkers, potentially clarifying tirzepatide’s renal protection and establishing MRI as a platform for assessing drug effects.

Among the biomarkers discussed, cystatin C–based eGFR and UACR have shown direct responses in tirzepatide clinical trials, whereas TNFR1/2, KIM-1, and NGAL are strong predictors of DKD progression but lack clinical evidence of change with tirzepatide. Comprehensive evaluation of these biomarkers in TREASURE-CKD and future trials could help define an optimal strategy for predicting and monitoring tirzepatide’s renal protective effects.

Clinical evidence for tirzepatide’s cardiovascular protection has grown. In the SUMMIT trial, it reduced the composite of cardiovascular death or worsening heart failure events in patients with heart failure with preserved ejection fraction (HFpEF) and obesity [11]. In SURPASS-CVOT, involving 13,299 patients with T2DM and atherosclerotic cardiovascular disease, tirzepatide was non-inferior to dulaglutide for cardiovascular death, myocardial infarction, or stroke, while also improving blood pressure, lipids, HbA1c, and body weight [149]. The following biomarkers may help predict or track these cardiovascular protective effects.

N-terminal pro-B-type natriuretic peptide (NT-proBNP) is a key heart failure biomarker, released by ventricular cardiomyocytes under wall stress, and is widely used to assess diagnosis, prognosis, and treatment response [150,151]. In SUMMIT, tirzepatide showed a trend toward reducing NT-proBNP at week 52 [12]. Since weight loss usually increases NT-proBNP, stable or decreased levels may indicate heart failure improvement [152]. In SURPASS-4, patients with T2DM and high cardiovascular risk who had higher baseline NT-proBNP experienced greater reductions with tirzepatide compared with insulin glargine [153], suggesting NT-proBNP may help stratify and predict cardiovascular benefits of tirzepatide.

High-sensitivity troponin T (hs-TnT) is the most sensitive marker of myocardial injury, reflecting low-grade damage common in HFpEF and predicting heart failure hospitalization and mortality [154]. In SUMMIT, tirzepatide significantly reduced hs-TnT versus placebo at week 52, with reductions evident from week 12 and maintained through week 52. The decrease correlated with hsCRP reduction, suggesting that tirzepatide’s anti-inflammatory effects may attenuate myocardial injury [12]. Thus, hs-TnT can monitor tirzepatide’s myocardial protective effects, especially in HFpEF.

High-sensitivity C-reactive protein (hsCRP) is a key biomarker of systemic inflammation and an independent predictor of cardiovascular risk, with levels > 3, 1–3, and <1 mg/L indicating high, intermediate, and low risk, respectively [155]. Agents that lower hsCRP, including statins and anti–IL-1β therapies, have been associated with improved cardiovascular outcomes [156,157]. Tirzepatide consistently reduces hsCRP across studies.

In SURPASS-4, hsCRP decreased by −38.0%, −44.2%, and −47.8% with tirzepatide 5, 10, and 15 mg, respectively, while remaining unchanged with insulin glargine (+0.6%), with many patients shifting to lower risk categories [158]. In SUMMIT, hsCRP similarly decreased by −38.8% with tirzepatide versus −5.9% with placebo [12]. These findings support hsCRP as a biomarker for monitoring tirzepatide’s anti-inflammatory and cardiovascular protective effects.

Tirzepatide also improves multiple cardiovascular risk–related biomarkers. In a post hoc phase 2 analysis, tirzepatide 10 and 15 mg doses significantly reduced intercellular adhesion molecule-1 (ICAM-1), chitinase-3-like protein-1 (YKL-40), growth differentiation factor-15 (GDF-15), and leptin at week 26 compared with placebo and dulaglutide [159]. Reductions in hsCRP, YKL-40, and ICAM-1 occurred within 4 weeks, whereas leptin declined more gradually, suggesting early anti-inflammatory and endothelial benefits partly independent of weight loss [159].

In lipoprotein-related biomarkers, tirzepatide dose-dependently reduced apolipoprotein B (ApoB) and apolipoprotein C-III (ApoC-III) and increased preheparin lipoprotein lipase [160]. Nuclear magnetic resonance analysis showed fewer large triglyceride-rich lipoproteins and small LDL particles, along with improved lipoprotein insulin resistance scores versus placebo and dulaglutide [160]. Changes in ApoC-III accounted for up to 22.9% of triglyceride variability independent of weight loss, suggesting direct effects of tirzepatide on lipid metabolism. These improvements in the atherogenic lipoprotein profile provide mechanistic support for the long-term cardiovascular protective effects of tirzepatide [160].

Among the cardiovascular biomarkers reviewed, hsCRP and hs-TnT showed significant changes with tirzepatide in the SUMMIT trial, while NT-proBNP serves as a complementary marker, reflecting heart failure improvement despite weight-loss–related upward trends. Endothelial and inflammatory markers (ICAM-1, YKL-40) and lipoprotein biomarkers (ApoB, ApoC-III) showed meaningful changes in phase 2 studies but require further validation in larger phase 3 trials.

## 9. Conclusions

Tirzepatide is a dual GLP-1/GIP receptor agonist that provides glycemic control, weight loss, and cardiovascular, hepatic, and renal protection. However, inter-individual variability highlights the need to identify response predictors to optimize efficacy and cost-effectiveness.

Predictors span five domains. Clinical predictors identified in SURPASS and SURMOUNT include tirzepatide dose, diabetes duration, β-cell function, baseline HbA1c, sex, age, race, concomitant medications, and early glycemic/weight responses, offering practical guidance for early treatment adjustment. Genetic predictors involve variants in *GLP1R*, *GIPR*, *ARRB1*, *TCF7L2*, *FTO*, *MC4R*, *KCNQ1*, *WFS1*, *SORCS1*, *FFAR1*, and *RAMP3*, affecting receptor binding, signaling, insulin secretion, and appetite. While effect sizes are modest, polygenic risk scoring may enhance prediction; most evidence comes from GLP-1RA studies, and tirzepatide-specific pharmacogenomics reflecting dual GIP/GLP-1 receptor activity remains to be explored.

Metabolomic biomarkers show that early decreases in BCAAs correlate with later HbA1c, HOMA2-IR, and proinsulin improvements, suggesting predictive value. Acylcarnitines, BHB, and bile acids are promising but lack direct clinical evidence. Proteomic and adipokine markers include FGF21, IGFBP-1/2, and the adiponectin/leptin ratio, reflecting weight loss and insulin sensitivity. Anti-inflammatory effects are captured by hsCRP and IL-6, supported by preclinical mechanisms such as NF-κB inhibition and M1-to-M2 macrophage polarization.

Organ-specific biomarkers show consistent responses: Pro-C3 and MRI-PDFF for liver, cystatin C–based eGFR and UACR for kidney protection. TNFR1/2, KIM-1, and NGAL predict DKD but lack tirzepatide-specific evidence; ongoing TREASURE-CKD may fill this gap.

Predictors can be stratified into three tiers (Table 3): (1) direct evidence in tirzepatide trials (clinical predictors, BCAAs, cystatin C–eGFR, UACR, Pro-C3, MRI-PDFF, IGFBP, adiponectin/leptin ratio), (2) strong disease-domain predictors requiring validation (TNFR1/2, KIM-1, NGAL, FGF21, genetic variants), and (3) mechanistically plausible but exploratory markers (acylcarnitines, BHB, bile acids, inflammatory cytokines). These predictors serve three distinct clinical roles: baseline predictors guide patient selection and initial dose titration; early on-treatment response indicators inform decisions regarding treatment adjustment; and treatment-monitoring biomarkers enable longitudinal assessment of efficacy and organ-specific benefits. These roles are summarized alongside the evidence tiers in Table 3.

Future directions include multi-omics integration, development of composite prediction models combining clinical, genetic, and molecular factors, and comprehensive evaluation in organ-protective studies using both imaging (multiparametric MRI) and biofluid (blood/urine) biomarkers.

Importantly, the factors reviewed in this article should be interpreted in a phenotype-specific manner rather than as universal predictors of an overall “tirzepatide response”. Clinical variables such as diabetes duration, baseline HbA1c, β-cell function, concomitant therapy, and early glycemic or weight changes are most directly relevant to glycemic and weight outcomes, primarily in T2DM and obesity. In contrast, hepatic biomarkers such as Pro-C3 and MRI-PDFF are more appropriately considered monitoring tools in MASH-related settings. Similarly, renal biomarkers such as UACR and cystatin C–based eGFR are more relevant for assessing renal outcomes in CKD- or albuminuria-related populations, while cardiovascular biomarkers, including hs-TnT and NT-proBNP, currently have the clearest relevance in HFpEF or high cardiovascular risk cohorts. Accordingly, generalizability across phenotypes should not be assumed without direct supporting evidence. To clarify phenotype-specificity and limits of extrapolation, representative predictors and biomarkers are summarized by clinical population and outcome domain (Table 4).

While the genetic, metabolomic, proteomic, adipokine, and organ-specific biomarkers reviewed here may, pending further validation, eventually inform clinical decision-making, tirzepatide-specific evidence for most remains limited. In contrast, readily accessible measures—such as HbA1c, body weight, UACR, and cystatin C–based eGFR—are supported by direct clinical data with tirzepatide. Accordingly, the prospective development and validation of a pragmatic, biomarker-driven algorithm for individualized tirzepatide therapy, anchored in these measures, represents a key priority for future research as clinical practice continues to evolve.

In conclusion, predicting tirzepatide responsiveness requires a multidimensional approach integrating clinical, genetic, and molecular factors. Whether systematic biomarker-based stratification will prove cost-effective relative to pragmatic trial-and-error dose titration remains an open empirical question requiring prospective, pre-specified evaluation. Although such stratification is not yet supported by sufficient evidence or cost-effectiveness data to justify routine clinical implementation, future studies may establish a framework to inform clinical decision-making.

## Figures and Tables

**Table 1:** Clinical Predictors of Tirzepatide Response.

Predictor	Target Outcome	Effect Size (OR/aOR, 95% CI)	Source Study	References
Higher tirzepatide dose	HbA1c and weight reduction	Dose-dependent effect (5/10/15 mg)	SURPASS 1–4; SURMOUNT 1–3	[8,9,10]
Shorter diabetes duration	HbA1c ≤ 6.5% at week 52	Independent predictor	SURPASS-4 post hoc	[16]
Higher baseline HOMA-β	HbA1c ≤ 6.5% at week 52; glycemic durability (years 2)	OR 1.34 (1.06–1.69) for years 2 maintenance	SURPASS-4 post hoc	[16]
Lower baseline HbA1c	HbA1c target attainment; ≥15% weight reduction	aOR 1.28 per 1% decrease (1.15–1.43) for ≥15% weight reduction	Pooled SURPASS 1–4; SURPASS-4 post hoc	[7,16]
Female sex	≥15% and ≥10% weight reduction	aOR 2.63 (2.19–3.17) for ≥15% weight reduction	Pooled SURPASS 1–4; SURPASS-4 post hoc	[7,16]
Younger age	≥15% weight reduction	aOR 0.94 per 5-year increase (0.90–0.99)	Pooled SURPASS 1–4	[7]
White or Asian race	≥15% weight reduction	Higher probability vs. other racial groups	Pooled SURPASS 1–4	[7]
Baseline metformin monotherapy	HbA1c target attainment; ≥15% weight reduction	aOR 1.77 (1.27–2.46) for ≥15% weight reduction	SURPASS-4 post hoc; pooled SURPASS 1–4	[7,16]
Sulfonylurea use at week 52	Glycemic durability (years 2)	OR 0.56 (0.37–0.85)	SURPASS-4 post hoc	[16]
Absence of baseline albuminuria	HbA1c ≤ 6.5% at week 52	Independent predictor	SURPASS-4 post hoc	[16]
Lower baseline FPG and non-HDL cholesterol	≥15% weight reduction	Significant in multivariate model	Pooled SURPASS 1–4	[7]
Early FPG response (≥20% reduction at week 4)	Greater HbA1c and FPG reduction at weeks 40–42	Early responders > non-early responders	SURPASS post hoc	[32]
Early weight response (≥5% reduction at week 8)	Greater weight loss and cardiometabolic improvement at weeks 40–42	Early responders > non-early responders	SURPASS post hoc	[32]

Abbreviations: aOR, adjusted odds ratio; CI, confidence interval; FPG, fasting plasma glucose; HbA1c, hemoglobin A1c; HDL, high-density lipoprotein cholesterol; HOMA-β, homeostatic model assessment of β-cell function; OR, odds ratio.

**Table 3:** Tirzepatide response predictors and biomarkers. This table is organized by evidence tier and clinical role. Each entry is classified as a baseline predictor (assessed prior to treatment initiation to inform patient selection), an early on-treatment response indicator (measured within the first 4–12 weeks to guide continuation or dose adjustment), or a treatment-monitoring biomarker (assessed longitudinally to evaluate efficacy and organ-specific outcomes). Evidence tiers are defined as follows: Tier 1—direct tirzepatide clinical trials; Tier 2—strong disease-domain evidence requiring tirzepatide-specific validation; Tier 3—mechanistic or preclinical rationale with limited clinical support.

Evidence Tier	Definition	Clinical Role	Biomarkers/Predictors	Key Supporting Evidence	Clinical Implication
**Tier 1**	Direct tirzepatide clinical evidence	Baseline predictor	Clinical predictors (dose, diabetes duration, HOMA-β, HbA1c, sex, age, race, medications)	SURPASS 1–4 and SURMOUNT 1–3 post hoc analyses [7,16,32]	Immediately applicable for initial treatment stratification
		Early on-treatment response indicator	Early reductions in fasting plasma glucose and body weight	SURPASS 1–4 and SURMOUNT 1–3 post hoc analyses [7,16,32]	Supports early response assessment and dose titration
		Early on-treatment response indicator	BCAAs (leucine, isoleucine, valine)	Pirro et al. 2022: early reduction (week 4) correlated with HbA1c and HOMA2-IR [79]	Potential early pharmacodynamic biomarker (weeks 4–12)
		Early response indicator and treatment-monitoring biomarker	Cystatin C–based eGFR	SURPASS-4, SURMOUNT-1, SUMMIT post hoc analyses [123,128,129]	Complementary renal biomarker; less affected by muscle mass artifact
		Early on-treatment response indicator and treatment-monitoring biomarker	UACR	SURPASS 1–5, SURMOUNT 1–2: dose-dependent reduction of 19–26% [13,123,128]	Primary renal biomarker for monitoring and risk stratification
		Treatment-monitoring biomarker	Pro-C3	T2DM biomarker studies and SYNERGY-NASH: consistent reduction [15,118]	Monitoring of hepatic fibrogenesis
		Treatment-monitoring biomarker	MRI-PDFF	SYNERGY-NASH: −46% to −57% reduction; ≥30% threshold validated [15,119,120]	Gold-standard noninvasive quantification of liver fat
		Early on-treatment response indicator and treatment-monitoring biomarker	IGFBP-1/2	Thomas et al. 2021: increased levels with tirzepatide vs. dulaglutide [6]	Marker of improved insulin sensitivity
		Early on-treatment response indicator and treatment-monitoring biomarker	Adiponectin-to-leptin ratio	Multiple studies: adiponectin ↑ and leptin ↓ with tirzepatide [6,103]	Indicator of adipose tissue remodeling and metabolic health
		Treatment-monitoring biomarker	hsCRP	SUMMIT: −38.8% vs. −5.9% placebo (*p* < 0.001); SURPASS-4: −38% to −48% reduction [12,158]	CV risk and systemic inflammation monitoring
		Treatment-monitoring biomarker	hs-TnT	SUMMIT: ETD −10.4% (*p* = 0.003); significant from week 12 through week 52 [12]	Monitoring myocardial injury (e.g., HFpEF)
		Treatment-monitoring biomarker	NT-proBNP	SUMMIT: trend toward reduction (*p* = 0.07); SURPASS-4: greater reduction with higher baseline (interaction *p* = 0.0312) [12,153]	Complementary biomarker for heart failure stratification
**Tier 2**	Strong disease-domain evidence requiring tirzepatide-specific validation	Baseline predictor	TNFR1/2	Joslin Kidney Study (2012); meta-analysis RR 2.51–3.23 for DKD progression [132,133,134]	High-priority candidates for validation (e.g., TREASURE-CKD)
		Treatment-monitoring biomarker	KIM-1	Sabbisetti et al., 2014: correlated with eGFR decline (r = 0.52); preclinical reduction observed [140,144]	Early detection of tubular injury
		Treatment-monitoring biomarker	NGAL	Meta-analysis: sensitivity 0.79–0.90, specificity 0.87–0.97 for DKD [147]	Monitoring tubular injury and renal protection
		Baseline predictor	FGF21	Le et al. 2023: attenuated GLP-1RA weight loss in liver-specific FGF21 knockout mice [94]	Potential determinant of weight loss variability
		Baseline predictor	Genetic variants (*GLP1R, GIPR, ARRB1,* etc.)	GWAS and pharmacogenomic studies; PRS approaches [35,45,72]	Future precision medicine strategies
		Early on-treatment response indicator + Treatment-monitoring biomarker	ICAM-1/YKL-40/GDF-15	Phase 2 studies: significant reduction at week 26; early changes (week 4) for ICAM-1, YKL-40 [159]	Endothelial and inflammatory CV risk monitoring; requires phase 3 validation
		Treatment-monitoring biomarker	ApoB, ApoC-III, LPIR score	Phase 2: dose-dependent reduction; ApoC-III explained 22.9% of TG variability independently of weight loss [160]	Monitoring atherogenic lipoprotein profile; requires validation
**Tier 3**	Mechanistic or preclinical rationale with limited clinical support	Treatment-monitoring biomarker	Acylcarnitines	Elevated in T2DM and IR; no consistent change with tirzepatide [79,80,81,82]	Requires tirzepatide-specific clinical validation
		Treatment-monitoring biomarker	β-Hydroxybutyrate	Inversely associated with IR; >0.5 mM linked to weight reduction [85,86]	Exploratory marker of metabolic flexibility
		Treatment-monitoring biomarker	Bile acid profiles	Preclinical studies: favorable shifts with tirzepatide [89]	Requires human clinical validation
		Treatment-monitoring biomarker	Inflammatory cytokines (IL-6, TNF-α, MCP-1)	Meta-analysis: reductions in hsCRP and IL-6; preclinical NF-κB pathway inhibition [108,109,110,111]	Monitoring anti-inflammatory effects

Abbreviations: ApoB, apolipoprotein B; ApoC-III, apolipoprotein C-III; ARRB1, beta-arrestin 1; BCAAs, branched-chain amino acids; CKD, chronic kidney disease; CV, cardiovascular; DKD, diabetic kidney disease; eGFR, estimated glomerular filtration rate; FGF21, fibroblast growth factor 21; GDF-15, growth differentiation factor-15; GIPR, glucose-dependent insulinotropic polypeptide receptor; GLP-1RA, glucagon-like peptide-1 receptor agonist; GLP1R, GLP-1 receptor; GWAS, genome-wide association study; HbA1c, hemoglobin A1c; HFpEF, heart failure with preserved ejection fraction; HOMA-β, homeostatic model assessment of β-cell function; hsCRP, high-sensitivity C-reactive protein; hs-TnT, high-sensitivity troponin T; ICAM-1, intercellular adhesion molecule-1; IGFBP-1/2, insulin-like growth factor binding protein-1/2; IL-6, interleukin-6; IR, insulin resistance; KIM-1, kidney injury molecule-1; LPIR, lipoprotein insulin resistance; MCP-1, monocyte chemoattractant protein-1; MRI-PDFF, magnetic resonance imaging–proton density fat fraction; NF-κB, nuclear factor kappa-B; NGAL, neutrophil gelatinase-associated lipocalin; NT-proBNP, N-terminal pro-B-type natriuretic peptide; Pro-C3, N-terminal propeptide of type III collagen; PRS, polygenic risk score; T2DM, type 2 diabetes mellitus; TG, triglyceride; TNF-α, tumor necrosis factor-alpha; TNFR1/2, tumor necrosis factor receptor 1/2; UACR, urine albumin-to-creatinine ratio; YKL-40, chitinase-3-like protein-1.

**Table 4:** Phenotype-Specific Interpretation and Generalizability of Representative Tirzepatide-Associated Factors. Representative clinical predictors and biomarkers are organized according to the clinical phenotype or population in which they are most relevant. This table distinguishes glycemic and weight-related factors from hepatic, renal, and cardiovascular response-monitoring biomarkers and clarifies where extrapolation across outcome domains is not supported by direct evidence.

Predictor/Biomarker	Most Relevant Phenotype/Population	Primary Clinical Role	Generalizability
Diabetes duration, baseline HbA1c, HOMA-β, concomitant therapy	T2DM	Baseline predictors of glycemic target attainment	Not established for predicting hepatic, renal, or HF-related outcomes
Female sex, younger age, early weight change	Obesity and/or T2DM	Predictors and early indicators of weight-loss response	Limited applicability to organ-specific outcomes
BCAAs	T2DM; metabolic response studies	Early on-treatment metabolic indicator	Promising for glycemic/metabolic response; not validated for hepatic, renal, or HF outcomes
Pro-C3, MRI-PDFF	MASH/hepatic steatosis	Hepatic response-monitoring biomarkers	Phenotype-specific; not baseline predictors of overall tirzepatide efficacy
UACR, cystatin C-based eGFR	CKD/DKD or albuminuric populations	Renal response-monitoring biomarkers	Renal-specific; should not be extrapolated to glycemic or hepatic response prediction
TNFR1/2, KIM-1, NGAL	CKD/DKD-related populations	Exploratory renal biomarkers	Strong disease-domain relevance, but limited direct tirzepatide-treated populations
hs-TnT, NT-proBNP, hsCRP	HFpEF or high cardiovascular risk populations	Cardiovascular/HF response-monitoring biomarkers	Most applicable in cardiovascular phenotypes; broader extrapolation remains uncertain
*GLP1R, GIPR, ARRB1* and other genetic variants	Incretin-based therapy or pharmacogenomic study populations	Exploratory candidate predictors	Population- and endpoint-specific effects remain uncertain; limited tirzepatide-specific clinical validation

Abbreviations: ARRB1, beta-arrestin 1; BCAAs, branched-chain amino acids; CKD, chronic kidney disease; DKD, diabetic kidney disease; eGFR, estimated glomerular filtration rate; HbA1c, glycated hemoglobin; HF, heart failure; HFpEF, heart failure with preserved ejection fraction; hsCRP, high-sensitivity C-reactive protein; hs-TnT, high-sensitivity troponin T; KIM-1, kidney injury molecule-1; MASH, metabolic dysfunction-associated steatohepatitis; MRI-PDFF, magnetic resonance imaging–proton density fat fraction; NGAL, neutrophil gelatinase-associated lipocalin; NT-proBNP, N-terminal pro-B-type natriuretic peptide; T2DM, type 2 diabetes mellitus; TNFR1/2, tumor necrosis factor receptor 1/2; UACR, urine albumin-to-creatinine ratio.

## Data Availability

No new data were created or analyzed in this study. Data sharing is not applicable to this article.

## Abbreviations

The following abbreviations are used in this manuscript:

AKI	Acute kidney injury
AKT	Protein kinase B
ALT	Alanine aminotransferase
ApoB	Apolipoprotein B
ApoC-III	Apolipoprotein C-III
APRI	AST to platelet ratio index
ARRB1	Beta-arrestin 1
AUROC	Area under the receiver operating characteristic curve
BCAAs	Branched-chain amino acids
BHB	Beta-hydroxybutyrate
BMI	Body mass index
BOLD	Blood oxygen level dependent
cAMP	Cyclic adenosine monophosphate
CKD	Chronic kidney disease
CREB	cAMP response element-binding protein
DKD	Diabetic kidney disease
ECM	Extracellular matrix
eGFR	Estimated glomerular filtration rate
ELF	Enhanced Liver Fibrosis
ERK	Extracellular signal-regulated kinase
ESKD	End-stage kidney disease
FFAR1	Free fatty acid receptor 1
FGF21	Fibroblast growth factor 21
FIB-4	Fibrosis-4 Index
FTO	Fat mass and obesity-associated protein
GDF-15	Growth differentiation factor-15
GIP	Glucose-dependent insulinotropic polypeptide
GLP-1	Glucagon-like peptide-1
GLP-1RAs	Glucagon-like peptide-1 receptor agonists
GPCR	G protein-coupled receptor
GPR40	G protein-coupled receptor 40
GWAS	Genome-wide association studies
HbA1c	Hemoglobin A1c
HDL	High-density lipoprotein
HFpEF	Heart failure with preserved ejection fraction
HOMA-β	Homeostatic model assessment of β-cell function
HOMA2-β	Homeostatic model assessment 2 of β-cell function
HOMA2-IR	Homeostatic model assessment 2 of insulin resistance
hsCRP	High-sensitivity C-reactive protein
hs-TnT	High-sensitivity troponin T
ICAM-1	Intercellular adhesion molecule-1
IGFBP-1/2	Insulin-like growth factor binding protein-1/2
IL-6	Interleukin-6
KCNQ1	Potassium voltage-gated channel subfamily Q member 1
KIM-1	Kidney injury molecule-1
LDL	Low-density lipoprotein
MASH	Metabolic dysfunction-associated steatohepatitis
MC4R	Melanocortin 4 receptor
MCP-1	Monocyte chemoattractant protein-1
MRI	Magnetic resonance imaging
MRI-PDFF	Magnetic resonance imaging–proton density fat fraction
mTOR	Mechanistic target of rapamycin
NAFLD	Non-alcoholic fatty liver disease
NF-κB	Nuclear factor kappa-B
NGAL	Neutrophil gelatinase-associated lipocalin
NT-proBNP	N-terminal pro-B-type natriuretic peptide
PRS	Polygenic risk scores
RAMP3	Receptor activity-modifying protein 3
SORCS1	Sortilin-related VPS10 domain-containing receptor 1
T1DM	Type 1 diabetes mellitus
T2DM	Type 2 diabetes mellitus
TCA	Tricarboxylic acid
TCF7L2	Transcription factor 7-like 2
TGR5	Takeda G protein-coupled receptor 5
TNF-α	Tumor necrosis factor-alpha
TNFR1/2	Tumor necrosis factor receptor 1/2
UACR	Urine albumin-to-creatinine ratio
WFS1	Wolframin ER transmembrane glycoprotein
YKL-40	Chitinase-3-like protein-1
